# Portal hypertension and bleeding esophageal varices caused by portal vein thrombosis in children: experience of a single center with emphasis on treatments and results

**DOI:** 10.1016/j.clinsp.2025.100801

**Published:** 2025-10-29

**Authors:** Ana Cristina Aoun Tannuri, Amanda Anacleto dos Santos Barsuttini, Nelson Elias Mendes Gibelli, Giovanna Pedreira, Maria Luiza Villela Corullon, Pedro Zanetta Brenner, Manoel Ernesto Peçanha Gonçalves, Silvia Regina Cardoso, Diamari Caramelo Ricci Cereda, Uenis Tannuri

**Affiliations:** Pediatric Surgery Division, Pediatric Liver Transplantation Unit and Laboratory of Research in Pediatric Surgery (LIM 30), Faculdade de Medicina da Universidade de São Paulo, São Paulo, SP, Brazil

**Keywords:** Portal vein thrombosis, Portal hypertension, Meso-rex bypass, Portosystemic shunts, Digestive endoscopy, Endoscopic treatment

## Abstract

Background and aim: Portal Vein Thrombosis (PVT) is the most common cause of Portal Hypertension (pH) in children. This study aimed to present the experience of a large university center by analyzing patients with portal hypertension caused by portal vein thrombosis in search of prognostic factors, types of treatments, and results.

Methods: We retrospectively analyzed 110 pediatric patients treated from 2000 to 2019, who were divided into two groups: endoscopic procedures (67 patients) and surgical assistance protocols (43 patients).

Results: Among the patients who underwent the surgical protocol, 22 underwent Meso-Rex Shunt (MRS) and 21 underwent other surgical techniques: distal splenorenal shunt (DSRS ‒ 11 patients), azigoportal dissociation with partial splenectomy (6 patients), and mesocaval shunt (4 patients). Five patients with meso‑Rex developed graft thrombosis (5/22, 22.72 %). There were no differences between the groups regarding endoscopic findings (first and last), no correlation with the need for surgery, and no definition of the initial endoscopic characteristics that could predict worsening disease. The only observed difference was the increase in platelet count in the surgical group (median 25.02 thousand/mL, interquartile interval 118.00), which was greater than that observed in the endoscopic group (-42.50 thousand/mL, interquartile interval 56, *p* < 0.001, Mann-Whitney test). Only umbilical catheterization could predict the need for exclusive endoscopic treatment.

Conclusions: As the evolution of children treated endoscopically was not different from that of children treated surgically, we conclude that a more conservative approach should be adopted, whenever possible.

## Highlights

• Portal Vein Thrombosis (PVT) is a cause of gastrointestinal bleeding in children.
• Results of endoscopic and surgical treatment are comparable.
• We concluded that a conservative approach should be adopted whenever possible.

## Introduction

Portal Vein Thrombosis (PVT) is the most common cause of Portal Hypertension in children (pH). The main consequences of this disease are gastrointestinal bleeding, esophageal or gastric varices, and splenomegaly. Other consequences arise years after treatment, such as ascites, hepatic dysfunction, portal biliopathy, failure to thrive, delayed sexual development, coagulopathy, and cognitive impairment associated with subclinical portosystemic encephalopathy.

Although universally known, the treatment of PVT in children is frequently discussed in current scientific literature. PVT can be divided into two categories: palliative and curative. The former focus of treatment may be based on potentially lethal complications, such as hemorrhage of gastric and esophageal varices, which are present in >70 % of patients.^1^ Among these, there are three groups of palliative treatments: 1) Primary prophylaxis, which is based on beta-blocker administration and endoscopic interventions, such as sclerotherapy and variceal ligation; 2) Acute bleeding management procedures; and 3) Secondary prophylaxis, which involves intervention on varices or the primary cause. Curative treatment involves surgery.

Surgical approaches aim to reduce the pressure in the portomesenteric venous system. These techniques can be divided into non-physiological shunts, which reduce blood flow to the portal system by deviating portal blood to the systemic circulation, and physiological shunts, in which all blood coming to the portal vein is received by the liver through bypass. Physiological venous deviations, such as Neso-Rex Shunt Surgery (MRS), are usually preferable because they produce better outcomes in terms of the metabolic profile and cause less encephalopathy and coagulopathy than other techniques.^1^^,^^2^

Endoscopic management of esophageal and gastric varices is a conservative alternative to surgical procedures, has well-documented benefits, and is considered the first line of therapy for secondary prophylaxis. In addition, long-term follow-up has shown that many cases tend to spontaneously and gradually reduce portal hypertension as children grow, decreasing both clinical and endoscopic manifestations.^3^, ^4^, ^5^ Similarly, there are no known clinical or endoscopic signs for predicting the evolution of the disease, or any signals indicating refractoriness after endoscopic treatment. Although surgical approaches present undeniable benefits in PVT management, the scientific community has not reached a consensus on when they should be considered the best therapeutic option during the course of the disease.

Our service is the largest pediatric university center in Brazil, with >1130 patients undergoing liver transplantation since 1989, along with other cases of noncirrhotic portal hypertension.^6^^,^^7^ Regarding the treatment of children with portal vein thrombosis without underlying hepatic disease, we have been indicating the meso‑Rex bypass as the first option for the treatment of children with portal vein thrombosis, according to the Baveno consensus recommendation.^8^ The current study aims to present our experience on treating these children since the year 2000, giving emphasis on the search of prognostic factors at presentation that indicates the need for a surgical approach.

## Methods

The present study is a retrospective analysis of the medical records of pediatric patients with portal vein thrombosis, without underlying hepatic disease, treated from 2000 to 2019 at the Pediatric Institute of the Clinical Hospital of the University of São Paulo Medical School (São Paulo, Brazil). During this time frame, 110 patients met the inclusion criteria and were analyzed. We reviewed the patients’ records for data regarding demographics, complications, and mortality. The Ethics Committee of our institution approved the study protocol (Programa Institucional de Bolsas de Iniciação Científica – PIBIC Brazil, process number 119,303/2019–1; Ethics Committee Process number CAAE 22,184,719.9.0000.0068, Opinion number 3996,128) and informed consent was obtained from the parents or legal guardians of all patients).

Surgical assistance protocol: Portal vein thrombosis was diagnosed through Doppler ultrasonography, and the main symptom presented by the patients was upper gastrointestinal bleeding. After the first episode of bleeding and the endoscopic diagnosis of esophageal or gastric varices, the patients underwent extensive research for underlying hepatic disease, comorbidity, or thrombophilia, as well as search for previous umbilical vein catheterization.

All the patients underwent an initial endoscopic examination. The utilized endoscopic techniques consisted in variceal sclerotherapy, rubber band ligation, and the application of intravascular tissue adhesives. Sclerotherapy involved injecting irritating substances into or around varicose veins, triggering an inflammatory reaction and inducing vascular thrombosis. Rubber band ligation involved endoscopically placing elastic rings on the varicose cord, causing its “strangulation”, followed by ischemic necrosis in the following days. Tissue adhesive injection involved the intravaricose injection of a substance derived from cyanoacrylic acid (N‑butyl‑2-cyanoacrylate), which, upon contact with blood, polymerizes and solidifies, obstructing the vessel lumen. The choice of the type of method depends on the anatomic aspect of the varices. Elastic variceal ligation and/or sclerotherapy were performed in the presence of esophageal or gastric varices. The frequency of subsequent endoscopic evaluations was determined by disease severity and bleeding risk.

Surgical treatment followed the specific indications listed in Table 1, according to a previous study by Superina et al.^9^

**Table 1:** Absolute and relative indications for the treatment of patients with portal hypertension caused by portal vein thrombosis.

Absolute	Relative
Refractoriness of varices despite adequate clinical and endoscopic treatment.	Symptomatic or activity-restricting splenomegaly.
Severe hypersplenism.	Large esophageal varices in poor access to health system settings.
Low platelet count (< 10,000 mm^3^).	
Infections or other recurrent life-threatening complications.	Neurocognitive tests suggesting portosystemic encephalopathy.
Symptomatic encephalopathy unresponsive to clinical therapy.	Portal biliopathy.
	Delayed sexual development unexplained by other causes.
Hepatopulmonary syndrome.	
Porto-pulmonary hypertension.	

**Fig. 1:**
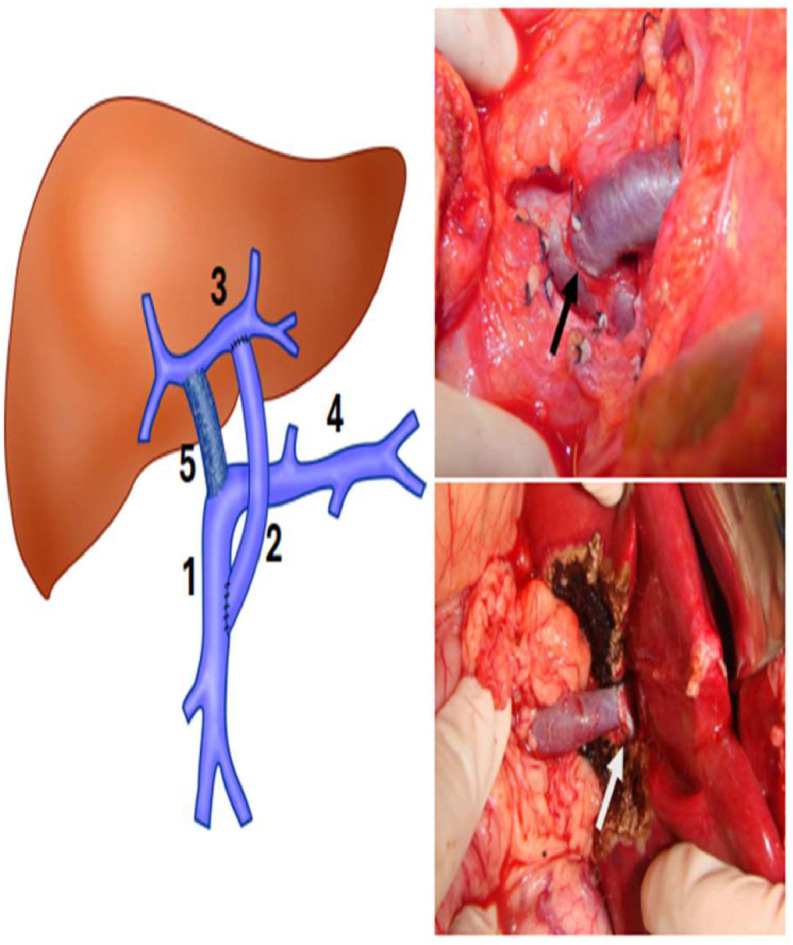
Left, schematic drawing showing MRS. (1) Superior mesenteric vein; (2) Venous graft; (3) Left portal vein in the Rex space; (4) Splenic vein; (5) Portal vein occluded by thrombosis. Right, on the top ‒ anastomosis between the venous graft and the superior mesenteric vein (black arrow). The white arrow indicates the anastomosis between the venous graft and left portal vein in the Rex recessus.

Patients with surgical indications underwent angiotomographic assessment of left portal branch patency and other intrahepatic portal vein circulation details. In the last 24-years, the MRS has been the first-choice approach. Briefly, through a small right subcostal incision, the liver bridge between segments 3 and 4 was divided, and the round ligament was followed into the Rex recessus to expose the left portal vein to confirm its permeability and if it was adequate for shunting. After this, the incision was prolonged, and all the terminal branches of the left portal vein were isolated carefully. A small lateral Satinsky clamp allowed good control of the left portal vein and its branches. The umbilical remnant was divided at its junction with the left portal vein, this venotomy was extended laterally and medially to achieve good extension to perform the anastomosis. After that, the inframesocolic space was accessed to expose the superior mesenteric vein following the middle colic vein, which was ligated near its junction with the superior mesenteric vein, allowing total exposure to clamp and perform the anastomosis without any difficulty. A venous autograft collected from the patient’s left cervical region (internal jugular vein) was anastomosed terminal-laterally. The graft was brought around the back of the antral-pyloric region and then through the transverse colon toward the left portal vein. Then, it was anastomosed end-to-side to the left portal vein. A gentle traction of the Satinsky clamp of the left portal vein facilitated the exposure to perform the anastomosis to the graft. All vascular anastomoses were performed via magnified loupes and continuous 7–0 absorbable monofilament sutures (polydioxanone) (Fig. 1). Three senior authors (NEMG, ACAT, and UT) performed or supervised all operations.^10^^–^^12^

Our second option of surgical procedure was the classical Warren shunt, in cases of preoperative or intraoperative diagnosis of splenic vein patency, according to previous experience at our center.^3^^,^^4^ The other surgical procedure was azigoportal dissociation, which was performed in cases of gastrointestinal bleeding, following a failed previously described procedure. Classical azigoportal dissociation with partial splenectomy, preserving the inferior pole of the spleen, was reserved for cases in which previous creation of a selective shunt had been unsuccessful or technically impossible. Partial splenectomy was performed by ligating all vascular branches close to the splenic parenchyma and preserving only the inferior vascular branches responsible for irrigation of the inferior pole of the spleen.

The patients were divided into two groups: endoscopic and surgical protocols. Patients in the endoscopic protocol group underwent only endoscopic procedures and had never undergone shunt or bypass surgery after diagnosis. The surgical protocol group included all patients who, at any point after the diagnosis, had undergone surgery for the correction of portal hypertension, regardless of having undergone endoscopic treatment before or after the procedure.

The collected variables included clinical and anthropometric data, initial clinical presentation, neonatal prematurity history, previous umbilical catheterization, disease duration and medical follow-up, number of gastrointestinal bleeding episodes, number of hospitalizations due to portal hypertension, type of surgery performed, need for more than one surgical approach, occurrence of bleeding after the last surgery, and peripheral blood platelet count (thousand/mL). All endoscopic procedures were performed by the same team of three professionals and were recorded for each patient's first and last endoscopy according to the following variables: history of previous bleeding, signs of previous bleeding, need for sclerotherapy or ligation, presence of esophageal or gastric varices, and presence of hypertensive gastropathy. Regarding esophageal varices and bleeding risk, our endoscopist group divided the patients into incipient (thin varices) and moderate or thick varices, based on their caliber and aspect, according to previous recommendations in the literature emphasizing the need to provide primary prophylaxis, even in cases of endoscopic patterns of high-risk varices.^13^ For patients in the surgical group, these findings were further sorted into preoperative or postoperative findings.

Multivariable logistic regression was performed considering the inclusion in the surgical protocol group, that is, the necessity for surgery. The variables analyzed were weight – underweight (< *Z*-score −2) and risk of being overweight, overweight, and obese (> *Z*-score +1), clinical findings at presentation of disease (gastrointestinal bleeding and splenomegaly), and history of umbilical vein catheterization. Additionally, statistical analyses using the Chi-Square method were performed between both groups regarding the endoscopic findings at presentation and at the time of the last endoscopy with results further scrutinized using the Bonferroni correction for multiple comparisons. Statistical analysis was performed using STATA13 software, and the significance value adopted was 95 %.

## Results

Among the 110 patients, 67 followed the endoscopic protocol and 43 followed the surgical protocol. Anthropometric, sociodemographic, clinical data, and clinical comparisons are presented in Table 2.

**Table 2:** Anthropometric data of groups and statistical analyses.

	Endoscopy group (*n* = 67)	Surgical group (*n* = 43)	p-value
**Sex**			0.815
Male	62.69 % (42)	60.47 % (26)	
Female	37.31 % (25)	39.53 % (17)	
**Race**			0.896
Black	4.48 % (3)	2.33 % (1)	
Mixed	11.94 % (8)	27.91 % (12)	
White	34.33 % (23)	67.44 % (29)	
Non specified	49.25 % (33)	2.33 % (1)	
**Age at diagnosis (years)**	4.73 ± 3.45	4.16 ± 3.15	0.395
**Weight at diagnosis (kg)**	18.21 (6.41 ‒ 77.72)	18.2 (6.09 ‒ 51.92)	0.636
**Height at diagnosis (cm)**	111.72 ± 25.63	113.02 ± 27.29	0.818
**Initial clinical manifestation**			0.976
Gastrointestinal bleeding	56.72 % (38)	53.49 % (23)	
Splenomegaly	31.34 % (21)	32.56 % (14)	
Abdominal mass or volume	2.99 % (2)	6.98 % (3)	
**Ultrasonography finding**			
Abdominal pain	7.46 % (5)	0.00 % (0)	
**Pallor**			
Non specified	1.49 % (1)	2.33 % (1)	
	0.00 % (0)	2.33 % (1)	
	0.00 % (0)	2.33 % (1)	
**Prematurity**			0.880
Yes	37.31 % (25)	20.93 % (9)	
No	55.22 % (37)	67.44 % (29)	
No data	7.46 % (5)	11.63 % (5)	
**Neonatal umbilical catheterization**			0.017
Yes	55.22 % (37)	30.23 % (13)	
No	44.78 % (30)	65.12 % (28)	
No data	0.00 % (0)	4.65 % (2)	
**Propranolol use**			0.480
Yes	73.13 % (49)	79.07 % (34)	
No	26.87 % (18)	20.93 % (9)	
**Hospitalization**	0.98 ± 1.50	4.13 ± 3.93	<0.001
**Bleeding episodes**	2.20 ± 2.17	4.40 ± 4.15	<0.001

The results of multivariate logistic regression considering the variables of interest for surgery need are presented in Table 3 as Odds Ratios, p-values, and 95 % Confidence Intervals.

**Table 3:** Results of multivariate logistic regression considering the variables for surgery indication.

	Odds Ratio	p-value	95 % Confidence Interval
**Underweight**	1.884627	0.402	0.4274396 ‒ 8.30952
**BMI Z-score > +1**	0.661682	0.424	0.2403465 ‒ 1.821633
**GI bleeding at presentation**	1.086461	0.908	0.2641838 ‒ 4.468095
**Splenomegaly at presentation**	1.263859	0.758	0.2846838 ‒ 5.610925
**Umbilical catheterization**	2.547722	0.046	1.015907 ‒ 6.389253

Pseudo R-squared 0.0452.

Among the patients who underwent surgical procedures, 31 needed only one surgical approach, and 12 needed multiple approaches to correct portal hypertension. The techniques used were the MRS in 22 patients, distal splenorenal shunt (DSRS ‒ Warren shunt) in 11 patients, and azigoportal dissociation with partial splenectomy in 6 patients. In four patients, a mesocaval shunt was performed. One of the patients developed voluminous hemorrhoids and anal bleeding, with good resolution achieved by performing an additional inferior mesenteric vein-internal iliac vein shunt.

Among the patients who underwent MRS surgery, three of them were diagnosed with stenosis at the portal-venous graft anastomosis and successfully treated by radio-intervention procedures, and five children (5/22 – 22.72 %) developed graft thrombosis. Two children presented with chylous ascites that spontaneously resolved within 3-weeks. Among the 43 surgical patients, 13 underwent their last surgery in the first year of follow-up, 11 of whom had undergone surgery in the first six months of follow-up. Seventeen children (39.53 %) still presented with gastrointestinal bleeding after the last surgical procedure due to the persistence of thrombocytopenia or gastrites.

Endoscopic examinations: The mean number of endoscopies performed in the endoscopic protocol group was 14.52 ± 9.27, whereas it was 13.37 ± 9.18 in surgical protocol group, 13.08 ± 8.64 among patients who underwent MRS surgery, and 13.82 ± 10.19 among those who underwent other procedures, respectively. The prevalence of large-caliber esophageal varices during the first endoscopy was 39.66 % in the endoscopic protocol group and 50.0 % in the surgical protocol group.

Endoscopic therapy was more common in the endoscopic protocol group than in the surgical protocol group (5.48 ± 4.31 and 3.49 ± 3.48, respectively; *p* = 0.01). Among the latter, 18.6 % (*n* = 8) underwent endoscopic therapy after the surgery, and all of them were subjected to procedures in addition to the MRS.

There was an increase in the number of endoscopies that did not require sclerotherapy or ligation of varices between the first and last endoscopies in both groups, especially among the surgical protocol patients (from 55.81 % to 93.94 %). In the last examination, only 2.33 % of the patients presented with high-risk esophageal varices in the endoscopic protocol group, and none in the surgical protocol group presented with high-risk esophageal varices.

The results of the comparative statistical analysis between protocols regarding endoscopic findings (first and last) and average age at the time of endoscopy (presented as the average in years ± standard deviation) are shown in Table 4. The p-value adopted for this analysis was updated using the Bonferroni correction, arriving at statistical significance of *p* = 0.036. In both groups, significant improvements were detected between the first and last endoscopy examinations when considering the caliber of esophageal varices, and high or moderate bleeding risk. The endoscopy group showed a higher grade of absent or low-risk varices at the end of follow-up, while the surgical group had reduced gastric varices.

**Table 4:** Comparative statistical analysis of groups regarding the endoscopic findings. Significant p-value considered after Bonferroni correction: *p* = 0.036.

		Endoscopy group		p-value	Surgical group		p-value
		67 patients			43 patients		
**Age at endoscopy (years)**		7.16 ± 4.14			6.57 ± 4.43		
		13.98 ± 4.67			13.15 ± 5.02		
**Characteristics**		**First endoscopy % of patients**	**Last endoscopy % of patients**		**First endoscopy % of patients**	**Last endoscopy % of patients**	
Esophageal varices		86.56	64.17	0.003	86.04	44.18	<0.001
Esophageal varices caliber	Thin or Incipient	20.89	47.76	0.002	23.26	41.86	0.106
	Moderate or Thick	61.19	10.44	<0.001	60.46	2.32	<0.001
Bleeding risk	Absent or Low	25.37	52.23	0.002	25.58	37.20	0.353
	Moderate or High	56.71	5.97	<0.001	51.16	2.32	<0.001
Hypertensive gastropathy		35.82	29.85	0.462	48.83	23.23	0.024
Gastric varices		65.67	49.25	0.05	83.72	32.55	<0.001

When we compared the endoscopy and surgical groups, we found no differences in these parameters (*p* = 0.498 for age at endoscopy, 0.266 for the presence of esophageal varices, 0.806 for caliber varices, 0.813 for bleeding risk, 0.227 for the presence of hypertensive gastropathy, and 0.061 for the presence of gastric varices). Finally, the increase in platelet count in the surgical group (median 25.02 thousand/mL, interquartile interval 118.00) was greater than that in the endoscopic group (median −42.50 thousand/mL, interquartile interval 56, *p* < 0.001, Mann-Whitney test). The follow-up period varied from 4 to 24 years (mean 13.5 years). No deaths occurred during the study period.

## Discussion

Although the pathogenesis of portal vein thrombosis in children and adolescents remains unknown, we present some forms of successful treatment based on the experience of our group in treating such patients. In the last century, from 1974 to 1984, we selected the DSRS as the procedure of choice for treating children with portal vein thrombosis, and forty-two children underwent it during this period.^4^ In 1985, Endoscopic Variceal ligation or Sclerotherapy (EVS) was introduced, and DSRS was replaced as the first therapeutic option in our service, with success rates greater than 95 %. Since then, endoscopic procedures have been the treatment of choice for patients with portal vein thrombosis and esophageal bleeding.^5^

After the description of MRS, we adopted this procedure as our first choice,^9^, ^10^, ^11^, ^12^ because it physiologically restores blood flow to the liver parenchyma. With this option, some authors have begun advocating the use of this technique for all cases of primary portal thrombosis, regardless of symptomatology and complications. However, long-term follow-up of these children subjected to prophylactic sclerotherapy or varices ligation provides evidence of spontaneous formation of collateral vessels and readjustment of portal circulation, reestablishing portal blood flow, and diminishing symptoms, especially gastrointestinal bleeding.(3–5,13) This classical evidence was universally known, even before the initial description of the MRS.^14^, ^15^, ^16^ Therefore, the actual indications of surgical treatment for children with PVT remain under debate, as there are no signs at diagnosis that could predict a more aggressive evolution or refractoriness to clinical and endoscopic therapy.

Our service is an important tertiary university center and the main pediatric liver transplantation center in Brazil, receiving children with liver diseases from all over the country and from South America. The patients were retrospectively studied due to the relatively low incidence of portal vein thrombosis in the pediatric population, which would make it difficult to publish a large series from a single center. The current study aimed to determine whether any criteria at presentation could predict a worse evolution and thus indicate the need for upfront surgical treatment. Comparisons between the two groups revealed no differences in the patients’ clinical or anthropometric data (Table 1). It was not possible to detect criteria at diagnosis that could point to more difficulties in the treatment of a child with portal vein thrombosis, and variables considered in the multivariate analysis showed no correlation with the need for surgery (pseudo-R-squared 0.0452), indicating their unreliability as predictors.

A secondary analysis was performed in both groups regarding the endoscopic findings at presentation and at the last endoscopy of each patient when comparing the groups. In both groups, we found a relatively high incidence of high-risk varices, and no definition of initial endoscopic characteristics that could predict a worse evolution.

In our study, only umbilical catheterization significantly predicted exclusive endoscopic treatment, corroborating the findings of Alberti et al. in 2013.^2^ In a previous study conducted at our institution, we described our initial experience with a MRS for cases of portal vein thrombosis in children and reported very low success rates for cases of previous umbilical catheterization.^10^ This may be a possible explanation for the current study’s findings, since there is a more selective indication for surgery in such cases and more insistence on endoscopic treatment in these children. Contrary to this knowledge, Lemoine et al. recently published a retrospective study indicating that previous umbilical vein catheterization is not a contraindication to MRS, as in their series, half of the patients were able to successfully undergo this procedure.^17^ However, it is important to note that the success rate of MRS was significantly higher in patients with no history of umbilical vein catheterization. Based on this observation and the current results, we still consider that the previous history of umbilical vein catheterization may be included as a possible limitation to the indication of MRS surgery.

In the present series, despite no findings suggesting the severity of the disease at the initial presentation, children who underwent surgical treatment were hospitalized more often, mostly because of gastrointestinal bleeding episodes (*p* < 0.001; Table 2). In addition, a more aggressive disease was present in these children early on, indicating the need for surgical treatment in the first six months of follow-up. Even after the first surgery, 12 of these patients required additional surgical procedures due to continued gastrointestinal bleeding episodes, suggesting a more aggressive disease in these patients. Five patients who underwent MRS surgery developed graft thrombosis, which led to maintenance of portal hypertension. Furthermore, these cases were perceived as more severe because almost 40 % of the children who underwent surgery still presented with gastrointestinal bleeding after the last surgical approach.

MRS should be considered as the primary surgical approach in children because it restores portal blood flow to the liver and has few complications. In the present study, this approach was performed in 22 children, allowing complete reversion of all signs of portal hypertension in 17 of them (77.27 %). As previously described, preoperative evaluation of the left portal branch via computerized angiotomography is very accurate and avoids the need for more invasive tests such as a retrograde portography. Finally, a possible criticism of our series would be the relatively high incidence of graft thrombosis. Certainly, this complication was caused by the fact that we did not select the cases for indicating the MRS. In addition, our follow-up time was long (period varied from 4 to 24-years, with a mean 13.5-years), in comparison to other series. In a recent publication from India, eighty-two shunts of all types were performed in children with portal vein thrombosis the MRS was possible in only 12 % of cases and the mean period of follow-up was 5-years.^18^

Among the postoperative complications, two children developed temporary chylous ascites, which spontaneously resolved. Additionally, stenosis at the portal venous-graft anastomosis was detected in three children, with good resolution achieved via radio-intervention procedures. These cases provide clear evidence of the importance of performing ultrasound examinations during the first months of postoperative follow-up to prevent complete shunt occlusion. For patients with surgical indications but no possibility of undergoing a MRS, we indicated a DSRS or azigoportal dissociation with partial splenectomy. We recommend partial splenectomy, even in cases of massive splenomegaly, to maintain immunological function and to prevent fulminant sepsis.^19^, ^20^, ^21^

The analyses of both groups showed that the evolution of endoscopic aspects during the study period presented significant improvements, considering the presence and caliber of the esophageal varices, bleeding risk, presence of hypertensive gastropathy, and presence of gastric varices. Finally, regarding the evolution of groups and the positive effects of the therapeutic methods (endoscopic or surgical treatment), no statistical differences between groups were detected (Table 4). Thus, conservative management of PVT cases with periodic endoscopic evaluation, opting for surgical treatment only in carefully selected patients, may produce satisfactory long-term results, with no mortality rate even in more aggressive cases.

Finally, we emphasize that the only difference observed between the studied groups was related to the peripheral blood platelet count. In fact, the surgical group presented a more significant improvement in this parameter, and this is a known response in patients who underwent MRS or other portosystemic shunts, according to other studies.^22^^,^^23^ Despite this advantage of the surgical groups of patients, our patients in the endoscopic group did not present relevant clinical problems related to decreased platelet count and hypersplenism. Based on the results of the current work, it is reasonable to consider that in the treatment of children with PVT, a more conservative approach should be adopted whenever possible. In future publications, we would suggest a prospective trial to validate the conservative approach herein proposed and identify the predictors of treatment failure in the situation of pediatric portal vein thrombosis and bleeding esophageal varices.

## Declarations

Ethics approval: The protocol for this article conforms to the provisions of the Declaration of Helsinki (1995) and has been approved by the Ethics Commission of Pediatric Institute and of the Clinical Hospital of the University of São Paulo Medical School (São Paulo, Brazil). Consent for publication: Not applicable.

Availability of data and material: The data sets used and/or analyzed during the current study are available from the corresponding author on reasonable request.

This Study follows the Observational Studies rules (STROBE Statement).

## Data access statement

All relevant data are within the paper and its Supporting Information files

## Ethical approval

The study protocol was approved by the Ethics Committee of Clinical Hospital of the University of São Paulo Medical School (São Paulo, Brazil) Ethics Committee Process number CAAE 22,184,719.9.0000.0068. The study protocol and informed consent were obtained from the parents or legal guardians of all the patients.

## Abbreviations

PVT, Portal Vein Thrombosis; pH, Portal Hypertension; DSRS, Distal Splenorenal Shunt; EVS, Endoscopic Variceal Sclerotherapy; MRS, Meso-Rex Shunt.

## Funding

This research was funded by a grant from the Programa Institucional de Bolsas de Iniciação Científica – PIBIC Brazil, process number 119303/2019–1

## CRediT authorship contribution statement

**Ana Cristina Aoun Tannuri:** Conceptualization, Data curation, Investigation, Methodology. **Amanda Anacleto dos Santos Barsuttini:** Funding acquisition, Investigation. **Nelson Elias Mendes Gibelli:** Data curation, Formal analysis. **Giovanna Pedreira:** Formal analysis, Methodology. **Maria Luiza Villela Corullon:** Formal analysis. **Pedro Zanetta Brenner:** Writing – review & editing. **Manoel Ernesto Peçanha Gonçalves:** Methodology. **Silvia Regina Cardoso:** Methodology. **Diamari Caramelo Ricci Cereda:** Methodology. **Uenis Tannuri:** Data curation, Supervision, Validation, Visualization, Writing – original draft, Writing – review & editing.

## Declaration of competing interest

The authors declare no conflicts of interest.
